# Case report: Diagnosis of a patient with Sifrim–Hitz–Weiss syndrome, development and epileptic encephalopathy-14, and medium chain acyl-CoA dehydrogenase deficiency

**DOI:** 10.3389/fped.2023.1230056

**Published:** 2023-09-04

**Authors:** Naim Zeka, Eris Zeka, Esra Zhubi, Ilir Hoxha

**Affiliations:** ^1^ Pediatric Clinic, University Clinical Center of Kosovo, Prishtina, Kosovo; ^2^ Faculty of Medicine, University of Prishtina, Prishtina, Kosovo; ^3^ Evidence Synthesis Group, Prishtina, Kosovo; ^4^ Janos Szentagothai Doctoral School of Neurosciences, Semmelweis University, Budapest, Hungary; ^5^ The Dartmouth Institute for Health Policy and Clinical Practice, Geisel School of Medicine at Dartmouth, Lebanon, NH, United States; ^6^ Research Unit, Heimerer College, Prishtina, Kosovo

**Keywords:** developmental and epileptic encephalopathy, genetic variants, medium-chain acyl-CoA dehydrogenase deficiency, neurodevelopmental delay, seizure, Sifrim–Hitz–Weiss syndrome

## Abstract

**Background:**

It is generally recognized that genetic metabolic disorders can result in neurological symptoms such as seizures, developmental delay, and intellectual disability. Heterogeneous clinical presentations make the diagnosis challenging.

**Case presentation:**

In this case report, we present a unique and complex genetic disorder observed in a female patient who exhibited three pathogenic gene variants in the *KCNT1, ACADM*, and *CHD4* genes. The convergence of these variants resulted in a multifaceted clinical presentation characterized by severe seizures of combined focal and generalized onset, metabolic dysfunction, and neurodevelopmental abnormalities. The identification and functional characterization of these gene variants shed light on the intricate interplay between these genes and the patient's phenotype. EEG revealed an epileptiform abnormality which presented in the inter-ictal period from the left frontal-central area and in the ictal period from the left mid-temporal area. The brain MRI revealed volume loss in the posterior periventricular area and parietal parenchyma, myelin destruction with no sign of hypoxic involvement, and left dominant enlargement of the lateral ventricles secondary to loss of central parenchyma. The patient was diagnosed through exome sequencing with Sifrim–Hitz–Weiss syndrome, development and epileptic encephalopathy-14, and medium-chain acyl-CoA dehydrogenase deficiency. An antiseizure medication regimen with valproic acid, levetiracetam, phenobarbital, and clonazepam was initiated. However, this led to only partial control of the seizures.

**Conclusion:**

Clinical follow-up of the patient will further define the clinical spectrum of *KCNT1*, *ACADM,* and *CHD4* gene variants. It will also determine the long-term efficacy of the treatment of seizures and the development of precision medicine for epilepsy syndromes due to gain-of-function variants. Special emphasis should be put on the role and importance of large-scale genomic testing in understanding and diagnosing complex phenotypes and atypical epileptic syndromes.

## Introduction

Neurodevelopmental disorders are a large group of disorders in which early events during brain development result in a broad and heterogeneous spectrum of clinical manifestations (1). Pathogenic gene variants and, consequently, inborn metabolic abnormalities represent a major disruptive cause in brain development (2, 3). More recently discovered, Sifrim–Hitz–Weiss is a multi-systemic neurodevelopmental disorder caused by heterozygous missense variants in chromodomain helicase DNA-binding protein 4 (*CHD4*) (4). *CHD4* encodes an ATP-dependent chromatin remodeler, an essential part of the nucleosome remodelling and histone deacetylation (NuRD) complex 3–8, which is widely expressed and acts primarily as a transcriptional repressor (4). This syndrome is characterized by developmental delay, intellectual disability, hearing loss, macrocephaly, distinct facial dysmorphisms, palatal abnormalities, ventriculomegaly, and hypogonadism (in males) (5, 6).

The genetic condition known as developmental and epileptic encephalopathy-14 is caused by a heterozygous variant in the *KCNT1* gene (7, 8). According to electrophysiological research, *KCNT1* variants lead to an imbalance between neuronal excitation and inhibition by increasing K+ currents in neurons. As a result a rare epileptic syndrome called epilepsy of infancy with migrating focal seizures (EIMFS) can be manifested (9). The abundant epileptiform activity from the gain-of-function variant in the *KCNT*1 gene interferes with brain development (10), resulting in cognitive delay and poor neurologic outcomes (11).

Medium-chain acyl-CoA dehydrogenase (MCAD) deficiency is an autosomal recessive genetic disorder caused by a homozygous variant in the *ACADM* gene (12). It is characterized by deficient levels of the medium-chain acyl-CoA dehydrogenase enzyme (12, 13). Therefore, in MCAD-deficient patients, not metabolized medium-chain fatty acids (MCFAs) accumulate in various tissues (13). Affected individuals are unable to generate enough energy from ketone bodies during periods of prolonged fasting or intense stress because of defective oxidation (14). Secondary symptoms of MCAD deficiency can develop from the damage of body tissues due to hypoglycemic events and hypoketotic hypoglycemia crisis might follow, which is clinically presented with nausea, fatigue, hepatomegaly, and liver dysfunction (15). Affected individuals may acquire as an inability to understand or use language (aphasia) and attention deficit disorder due to damage to the brain. These symptoms may progress to encephalopathy, seizures, coma, and even death (16, 17).

We report a case of a patient diagnosed through genetic testing with all three conditions, i.e., Sifrim–Hitz–Weiss syndrome, development and epileptic encephalopathy-14, and medium chain acyl-CoA dehydrogenase deficiency.

## Case presentation

We report the case of a four-year-old female patient who was presented at the pediatrics clinic at the University Clinical Centre of Kosovo with recurrent seizures and developmental delay. The patient was born on July 2018 at the gynecology and obstetrics clinic at the University Clinical Centre of Kosovo with an Apgar Score of 9/10. She was born at term, by vaginal delivery, and after a normal and uneventful pregnancy. The body weight at birth was 3,250 grams, the body height was 53 centimeters, and the head circumference was 34 centimeters. The patient was the first child from the first pregnancy of non-consanguineous parents. She had no history of perinatal asphyxiation, encephalitis, head injury, or febrile seizures. The parents denied the use of any medications. Her family history was negative for any neurological or genetic illnesses. An echocardiography was performed six days after the birth, and aberrantly inserted chorda tendineae in the left ventricle were observed.

In the third month of life, the patient experienced clusters of seizures of combined focal and generalized onset, which predominately occurred during the day. Seizures were generally brief, lasting from several seconds to a minute. They included tonic-clonic activity with motor activity being confined to one part of the body, migrating to another part of the body, and commonly becoming generalized seizures. In the beginning, the patient experienced one to two cluster of seizures weekly, which persisted in the following days at a higher frequency. The patient was admitted to the pediatric clinic in the University Clinical Centre of Kosovo. In a physical examination a relative microcephaly and a bilateral decreased muscle tone were evident.

**Figure 1:**
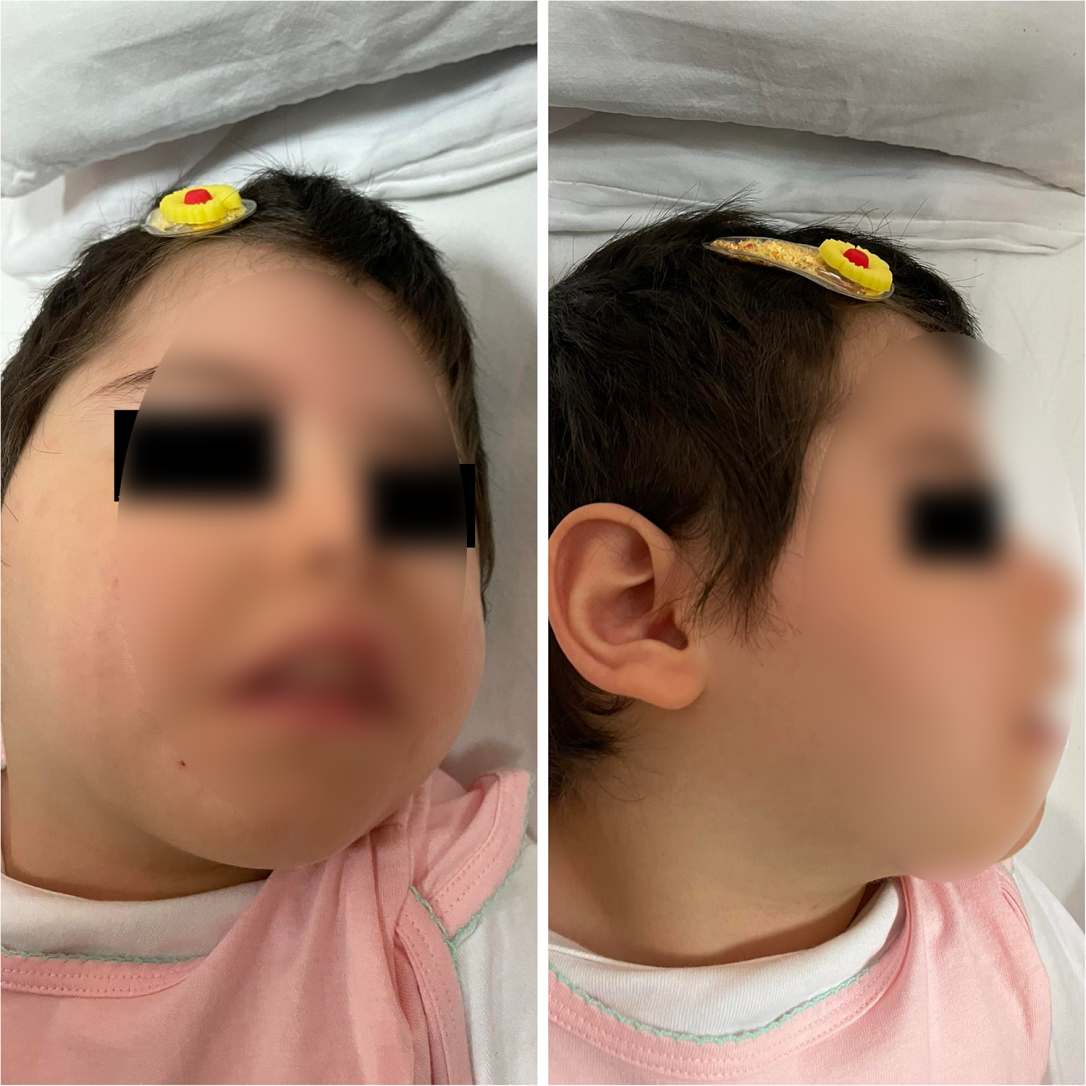
Facial dysmorphisms and microcephaly of the patient.

At the age of fifteen months, she was admitted to the neurology department of pediatrics clinic in the University Clinical Centre of Kosovo. In physical examination, a relative microcephaly, facial dimorphism (Figure 1), including micrognathia and slopping forehead, a weakness of the limbs, intellectual disability, speech delay, inability to stay seated, and inability to keep the head straight and up were observed. Moreover, the neurological exam revealed a bilateral decreased muscle tone and decreased reflex response of the limbs. At the age of 28 months the head circumference measurement was under the third percentile.

## Diagnostic assessment and therapeutic intervention

### Electroencephalogram (EEG)

**Figure 2:**
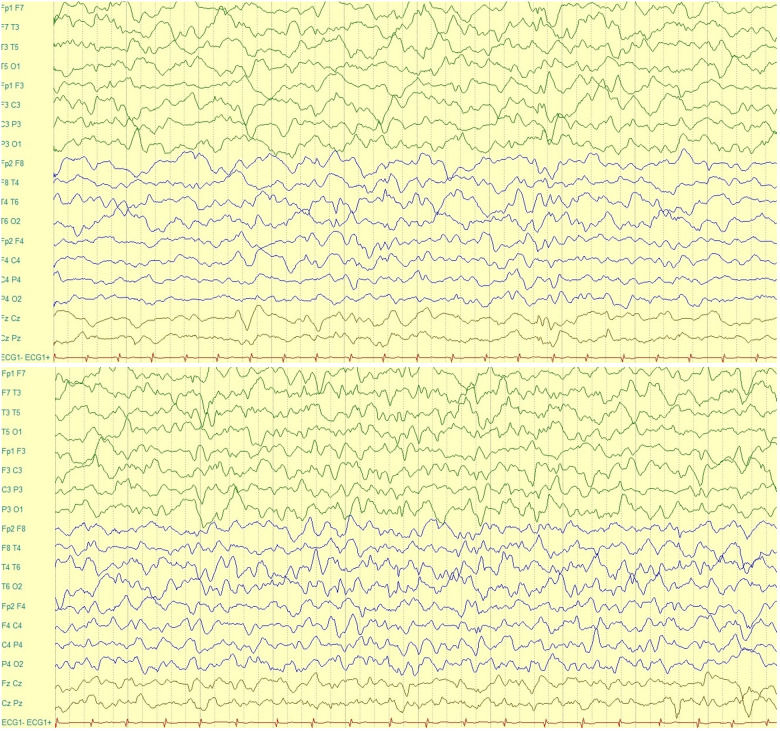
Electroencephalogram (EEG) of the patient.

An electroencephalogram (EEG) during wakefulness (Figure 2) was performed and it showed regular basic electro-cerebral activity, with predomination of alpha and teta rhythm and specific elements in both hemispheres. An EEG during sleep was performed, and it showed basic activity, which considering patient's age, was not well developed and there was a slower activity in the left hemisphere compared to the right one. In both hemispheres, sharp waves were observed. Moreover, the amplitude of waves of the left hemisphere were of high and low-frequency. A high-amplitude trunk was observed, which began from the frontal-central area on the left. It did not spread and had a slow-sharp activity. An epileptic activity that began in the ictal period is seen, which was built up in the mid-temporal area and lasted approximately 60 seconds. In conclusion, this EEG revealed an epileptiform abnormality which presented in the inter-ictal period from the left frontal-central area and in the ictal period from the left mid-temporal area.

### Magnetic resonance imaging (MRI) of the brain

**Figure 3:**
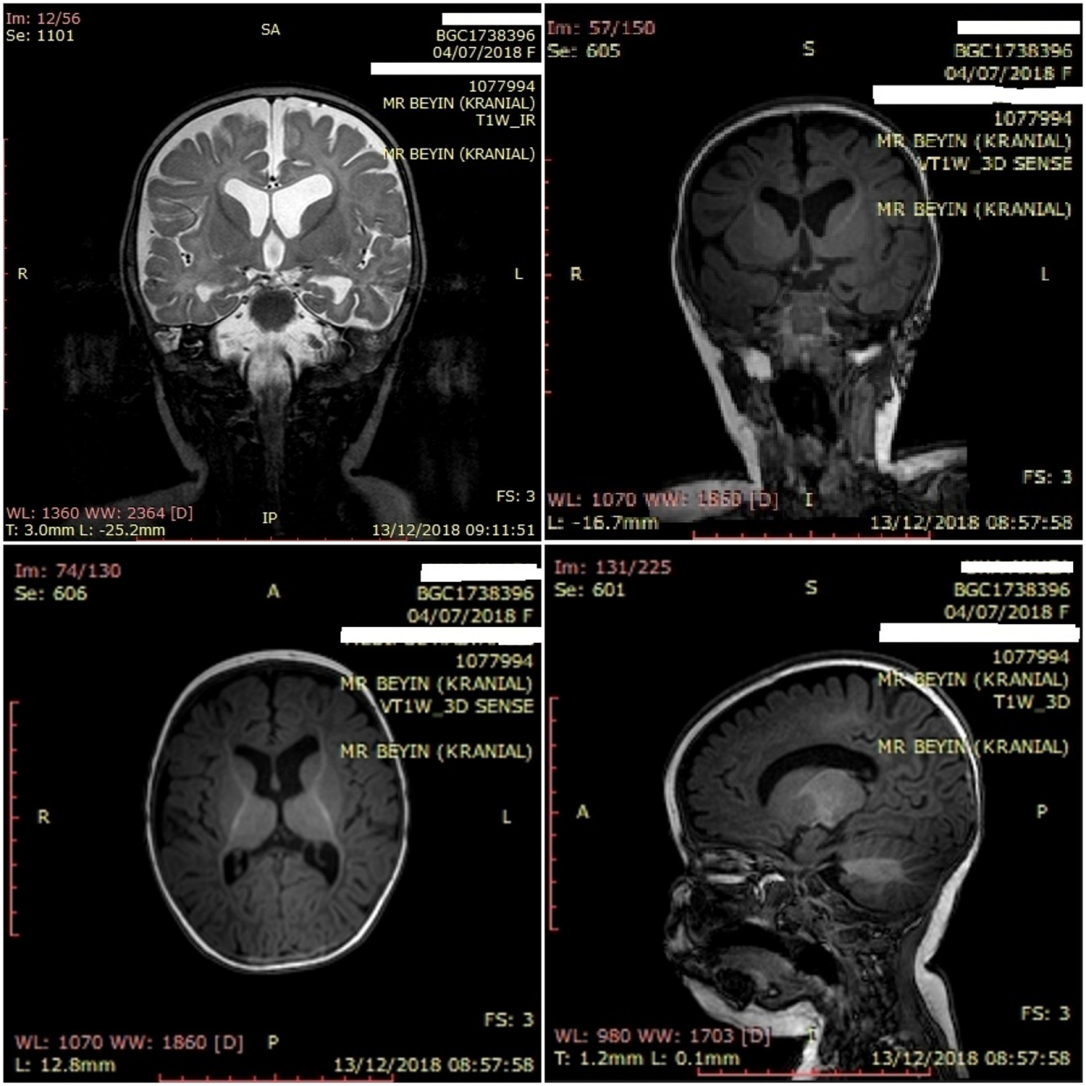
Brain magnetic resonance imaging (MRI) of the patient.

The patient underwent a brain MRI (Figure 3), which showed volume loss in the posterior periventricular area and parietal parenchyma and obvious myelin destruction with no sign of hypoxic involvement (gliosis). Left dominant enlargement of the lateral ventricles secondary to loss of central parenchyma was also observed. In contrast, bulbus, pons, mesencephalon, both cerebellar hemispheres, and vermis parenchyma were normal. The development of cerebral myelin was paracentral and the major cerebral vascular structures were patent. Cerebral sulcus pattern and cortex thickness were normal, and cortical dysplasia was absent.

### Exome sequencing (ES) analysis report

This patient's symptoms and clinical signs did not allow us to pinpoint a potential clinical diagnosis. Therefore, an Exome sequencing (ES) analysis was performed. While targeted genetic testing focuses on a single gene or a limited number of predetermined genes, the ES analysis simultaneously examines all protein-coding regions in the genome (exons). The DNA obtained from the biological material of the patient was enriched using the “QIAseq Human Exome” kit to cover all exome regions in humans (targeting 99% of the exonic regions including CCDS, RefSeq and renecode databases). The enriched library is sequenced to an average of 100X coverage on the QIANGEN next generation sequencing platform. The reference genome used in the study is GRCh37/hg19. In the bioinformatics analysis part, IDT WES Copy of Identify Variants Exome v1.1.1 workflow recommended by QIAGEN was used. QIAGEN Ingenuity Variant Analysis (IVA) and QIAGEN Clinical Insight (QCI) were used for variant interpretation and analysis. All variants in the panel with a reading depth of 10x, allele fraction 1% and quality score higher than 10% were examined. All disease-associated variants reported in the HGMD®, ClinVar or CentoMD® databases, as well as variants with a frequency of less than 1% in population databases such as ExAc, 1,000 Genomes gnomAD, and NHLBI ESP were also included. In addition, it was examined with the variants found in QIAGEN's own database (Allele Frequency Community). In the evaluation of the data obtained, +/−10 base pairs of intronic regions were also included in addition to the coded exonic regions.

The c.101-1G>C variant in the *CHD4* gene was detected in a heterozygous state. *CHD4* gene is located on chromosome 12 (12p13.31) and consists of 39 exons. Heterozygous variants in the *CHD4* gene are associated with Sifrim–Hitz–Weiss syndrome. The c.244dupT variant in the *ACADM* gene variant was detected as a homozygous state. This causes the production of shorter proteins due to the replacement of tryptophan with leucine at position 82. *ACADM* gene is located on chromosome 1 (1p31.1) and consists of 12 exons. Homozygous variants in the *ACADM* gene are associated with medium chain acyl-CoA dehydrogenase deficiency. The c.2665G>A variant of the *KCNT1* gene was detected as a heterozygous state, which causes the alanine amino acid at position 934 to be converted to threonine amino acid (missense variant). The *KCNT1* gene is located on chromosome 9 (9q34.3) and consists of 31 exons. Heterozygous variants in the *KCNT1* gene are associated with developmental and epileptic encephalopathy-14.

### Laboratory examinations and therapeutic intervention

Auxiliary examinations, including routine blood examinations, serum biochemical examination, cerebrospinal fluid (CSF) examination, urine and stool examination, and autoimmunity antibodies testing were unremarkable. Blood amino acids and acyl-carnitine analyses for inherited metabolic diseases, and urine organic acids were unrevealing at the first admission to our clinic. Further tests showed no fluid or electrolyte imbalance. Testing for toxoplasmosis, rubella, cytomegalovirus, and herpes simplex virus, and their respective antibodies were negative as determined by PCR and ELISA. However, after the thyroid function examination, our patient was diagnosed with congenital hypothyreosis, and treatment with Levothyroxine sodium (15 mcg/kg/day orally) was initiated.

A treatment regimen which consisted of multiple anti-seizure medications, such as valproic acid (450 mg/daily), levetiracetam (400 mg/daily), phenobarbital (50 mg/daily), and clonazepam (1.5 mg/daily) was initiated. Despite the regular administration and titration to the optimal dosage of the antiseizure medications, only partial control of the seizures was achieved, and the patients continued to experience tens of seizures per week. Moreover, our patient experienced a severe intellectual delay, failed to meet the developmental milestone for her age, and was frequently hospitalized in our clinic due to frequent seizures and respiratory distress.

## Discussion

In this study, we presented the case of a patient who experienced seizures of combined focal and generalized onset and developmental delay. Since there was an overlap of different symptoms and conditions, it was difficult to pinpoint a specific clinical diagnosis. Therefore, the ES analysis, which simultaneously examines all protein-coding regions in the genome (exons), was performed. Gene variants associated with Sifrim–Hits–Weiss syndrome, developmental and epileptic encephalopathy-14, and deficiency of medium chain of acyl-CoA dehydrogenase were found.

Sifrim–Hitz–Weiss syndrome is a recently described multi-systemic neurodevelopmental disorder, and a few numbers of patients exhibiting *de novo* pathogenic *CHD4* variants, causative of Sifrim–Hits–Weiss syndrome, have been reported to date (4). Weiss et al. initially reported five individuals with a form of syndromic intellectual disability that carried *de novo* missense variants in the *CHD4*, responsible for intellectual disability syndrome with distinctive facial dysmorphism (5). Further studies on the pathogenic variants of the *CHD4* gene associated the respective variants to clinical manifestations such as developmental delay, speech delay, and usually mild-to-moderate intellectual disability (4). Other clinical manifestations included generalized hypotonia of infancy, macrocephaly or relative microcephaly, congenital heart defects, skeletal and limb anomalies, hypogonadism (in males), ophthalmologic abnormalities, hearing impairment, and moyamoya disease with congenital or infantile stroke. Brain MRI findings include mild-to-moderate ventriculomegaly, Chiari type 1 malformation, hydrocephalus requiring shunting, thin corpus callosum and syringomyelia (4, 5).

Our patient manifested decreased muscle tone and decreased reflex response of the limbs. Moreover, an echocardiography was performed six days after the birth of the patient, and aberrantly inserted chorda tendineae in the left ventricle were observed. A recent study by Lui et al. presented four cases of heterozygous missense *CHD4* variants in four unrelated families, including two *de novo* variants (c.1597A > G/p.K533E and c.4936G > A/p. E1646K) and two inherited variants with co-segregation within the families (18). These gene variants were manifested in phenotype with childhood idiopathic epilepsy (15) and sinus arrhythmia (17). Likewise, congenital heart defects due to *de novo* heterozygous variants were identified by exome sequencing (18, 19). Table 1. presents the clinical characteristics of our case as compared to previously reported clinical characteristics of Sifirm-Hitz-Weiss syndrome.

**Table 1:** The clinical characteristics of the patient.

Clinical characteristics of previously reported SHW cases	Clinical characteristics of our reported SHW case
Developmental delay	+
Speech delay	+
Mild-to-moderate intellectual disability	+
Hypotonia and motor delay	+
Macrocephaly	−
Microcephaly	+
Abnormal brain imaging (ventriculomegaly, hydrocephalus requiring shunting, Chiari 1 and a thin corpus callosum)	+
Congenital heart defects (septal defects, conotruncal anomalies, and valve anomalies)	+
Moyamoya disease	−
Conductive and/or sensorineural hearing loss	−
Skeletal and limb anomalies (vertebral fusion and carpal/tarsal coalition, syndactyly, polydactyly)	−
Ophthalmic abnormalities (strabismus, hypermetropia, astigmatism)	−
Cryptorchidism and/or a micro-phallus	Not applicable
Hypogonadism in males	Not applicable
Dysmorphic features (broad forehead, squared face, periorbital fullness, widely spaced eyes, short nose and small or dysmorphic ears)	+

Another pathogenic variant found through the exome sequencing (ES) was the c.244dupT variant in the *ACADM* gene variant, responsible for the medium chain acyl-CoA dehydrogenase deficiency. Inherited deficiency of medium-chain of acyl-CoA dehydrogenase is characterized by intolerance to prolonged fasting, recurrent episodes of hypoglycemic coma with medium-chain dicarboxylic aciduria, impaired ketogenesis, and low plasma and tissue carnitine levels (19, 20). Symptomatic patients remained undiagnosed until a metabolic crisis. Asymptomatic patients were identified by neonatal mass screening or by sibling screening (21). Most of the symptomatic cases developed metabolic crises associated with hypoglycemia triggered by common infections and prolonged fasting (14). Usually, the clinical presentation of MCAD deficiency is related to fasting and increased metabolic stress, precipitating acute symptoms such as drowsiness or lethargy that may develop into a coma or even sudden death (22–24).

During the course of the disease, the patient manifested an arrest in psychomotor development in addition to recurring seizures and epileptiform activity in EEG. A pathogenic variant of the *KCNT1* gene was detected as a heterozygous state through exome sequencing, which is responsible for developmental and epileptic encephalopathy-14 (25). *KCNT1* encodes a sodium-activated potassium (KNa) channel that is highly expressed in the nervous system (26), and it is thought to regulate hyperpolarization following repetitive firing. *KCNT1* is also related to cardiac problems and leads to sudden unexpected death in epilepsy (SUDEP) (27).

A recent study evaluated the phenotypic aspects of a multi-ethnic cohort of *KCNT1* gene-related developmental and epileptic encephalopathy-14. Two-thirds of twenty-seven children involved in the study experienced epilepsy of infancy with migrating focal seizures (28). Another study by Ohba et al. concluded that *KCNT1* variants presented with ictal discharges on EEG that arise randomly from various areas of both hemispheres and migrate from one brain region to another, with or without clinical seizures (29). The disorder presents as “malignant migrating partial seizures of infancy” (30). Developmental plateauing upon onset is commonly exhibited in patients with EIMFS (31). Neurodevelopmental outcome was markedly impaired among 31 patients who experienced *KCNT1*-related severe early-onset epilepsy (31). The majority of the cases showed abnormal MRI findings, such as cortical development malformation, delayed myelination, thin corpus callosum, and brain atrophy (32).

The characteristic phenotype of individuals with *CHD3* and *CHD8* gene variants overlaps with the reported *CHD4* gene variants phenotype (33, 34). Blok et al. reported that *CHD3* variants presented with intellectual disability, macrocephaly, ventriculomegaly, undescended testicles, and similar facial features as seen in Sifrim–Hitz–Weiss syndrome (33). Patients with *CHD8* gene variants also exhibit a neurodevelopmental disorder characterized by overall excessive growth, developmental delays, intellectual disability, autism spectrum disorder, neuropsychiatric problems, sleep disturbances, and gastrointestinal issues (34).

Similarly, individuals harbouring homozygous *SCN1B* variants present with early infantile epileptic encephalopathy 52 (EIEE52), a rare, severe developmental and epileptic encephalopathy featuring infantile onset refractory seizures followed by developmental delay as seen in individuals harbouring the *KCNT1* variants (35). Early detection of the causative gene will enable the development of new drugs specifically targeting mutated proteins and selectively addressing pathogenic mechanisms, and therefore open new scenarios for personalized therapeutic approaches (36). Other metabolic disorders can exhibit similar clinical characteristics to the *ACADM* gene variant, responsible for the medium-chain acyl-CoA dehydrogenase deficiency (37, 38). Early infantile epileptic encephalopathy, caused by biallelic variants in *ARV1*, encoding a transmembrane protein of the endoplasmic reticulum with a pivotal role in glycosylphosphatidylinositol (GPI) biosynthesis, showed psychomotor delay, hypotonia, early onset refractory seizures followed by regression and specific neuroimaging features (37). Similarly, developmental and epileptic encephalopathy due to inosine triphosphate pyrophosphatase (ITPase) deficiency showed profound developmental delay, microcephaly, and refractory epilepsy followed by neurodevelopmental regression (38).

The case report possesses several strengths that contribute to its significance and clinical relevance. Firstly, the report provides a comprehensive and detailed description of the patient's medical history, presenting a thorough understanding of the clinical manifestation and progression. The inclusion of extensive genetic analysis and diagnostic tests further strengthens the validity of the findings. Moreover, the report highlights the exceptional nature of the case, emphasizing the rarity and uniqueness of the identified genetic variations. However, a limitation of the study is that it relies on a single patient, which limits the generalizability of the findings to a broader population. Additionally, the lack of available data on the long-term outcomes and response to treatment hinders a comprehensive understanding of the disorder's prognosis and therapeutic options. Despite these limitations, this case report serves as a valuable contribution in providing a foundation for further research and exploration in the field.

## Conclusion

In this study, we presented the detailed clinical features and genetic analysis of one patient with heterozygous variants in the *KCNT1* gene associated with “developmental and epileptic encephalopathy-14”, homozygous variants in the *ACADM* gene associated with “acyl-CoA dehydrogenase, medium chain, deficiency of”, and heterozygous variants in the *CHD4* gene associated with “Sifrim–Hitz–Weiss syndrome”.

Future studies on *KCNT1, ACADM*, and *CHD4* gene variants will help to make genotype-phenotype correlations clearer. Clinical follow-up of additional patients will further define the clinical spectrum of *KCNT1, ACADM*, and *CHD4*-related neurodevelopmental delay, the long-time efficacy of treatment of seizures, and the development of underlying precision medicine for epilepsy syndromes due to gain-of-function variants. Genetic analysis is essential to provide a precise diagnosis, which is crucial for managing the patient and the family members.

In conclusion, this case expands our understanding of the intricate interplay between genetics and disease, emphasizing the importance of continued research and exploration in the field. The rarity of this genetic configuration further underscores the need for comprehensive genetic profiling and personalized approaches in diagnosing and treating complex medical conditions.

## Data Availability

The original contributions presented in the study are included in the article/supplementary material, further inquiries can be directed to the corresponding author.
